# Stratifying patients using fast multiple kernel learning framework: case studies of Alzheimer’s disease and cancers

**DOI:** 10.1186/s12911-020-01140-y

**Published:** 2020-06-16

**Authors:** Thanh-Trung Giang, Thanh-Phuong Nguyen, Dang-Hung Tran

**Affiliations:** 1 VNU University of Engineering and Technology, Hanoi, Vietnam; 2 TayBac University, Son La, Vietnam; 3 Life Sciences Research Unit, Belval, University of Luxembourg, Luxembourg City, Luxembourg; 4 Megeno S.A., Belval, Esch-sur-Alzette, Luxembourg; 5 Hanoi National University of Education, Hanoi, Vietnam

**Keywords:** Patient stratification, Alzheimer’s diseases, Cancers, Multiple kernel learning, High dimensional data space, Dimension reduction

## Abstract

**Background:**

Predictive patient stratification is greatly emerging, because it allows us to prospectively identify which patients will benefit from what interventions before their condition worsens. In the biomedical research, a number of stratification methods have been successfully applied and have assisted treatment process. Because of heterogeneity and complexity of medical data, it is very challenging to integrate them and make use of them in practical clinic. There are two major challenges of data integration. Firstly, since the biomedical data has a high number of dimensions, combining multiple data leads to the hard problem of vast dimensional space handling. The computation is enormously complex and time-consuming. Secondly, the disparity of different data types causes another critical problem in machine learning for biomedical data. It has a great need to develop an efficient machine learning framework to handle the challenges.

**Methods:**

In this paper, we propose a fast-multiple kernel learning framework, referred to as fMKL-DR, that optimise equations to calculate matrix chain multiplication and reduce dimensions in data space. We applied our framework to two case studies, Alzheimer’s disease (AD) patient stratification and cancer patient stratification. We performed several comparative evaluations on various biomedical datasets.

**Results:**

In the case study of AD patients, we enhanced significantly the multiple-ROIs approach based on MRI image data. The method could successfully classify not only AD patients and non-AD patients but also different phases of AD patients with AUC close to 1. In the case study of cancer patients, the framework was applied to six types of cancers, i.e., glioblastoma multiforme cancer, ovarian cancer, lung cancer, breast cancer, kidney cancer, and liver cancer. We efficiently integrated gene expression, miRNA expression, and DNA methylation. The results showed that the classification model basing on integrated datasets was much more accurate than classification model basing on the single data type.

**Conclusions:**

The results demonstrated that the fMKL-DR remarkably improves computational cost and accuracy for both AD patient and cancer patient stratification. We optimised the data integration, dimension reduction, and kernel fusion. Our framework has great potential for mining large-scale cohort data and aiding personalised prevention.

## Background

Patient stratification has widespread biomedical and clinical applications, including diagnosis, prognosis, and treatment response prediction. A clinically useful prediction algorithm should be accurate, generalizable, be able to integrate diverse data types, and handle sparse data [1–5]. To achieve effective personalised medicine, patient stratification models are essentially required for all of diseases. Amongst the most emerging diseases, cancer and Alzheimer’s disease (AD) have been attracted a lot of research due to the severity, the complication and the high prevalence.

Alzheimer’s disease is a neurological disorder in which the death of brain cells causes memory loss and cognitive decline. Aging is the primary cause of AD, however, there are several other reasons related to lifestyle, such as physical inactivity, obesity, unhealthy diets, alcohol abuse, etc. [6]. Among AD’s phases, Mild Cognitive Impairment (MCI) are a critical phase because patients with MCI have higher risks for late stage of AD or other dementias. Stratifying AD patients in the early stage is crucial, so that we identify cases whose MCI signs may potentially be converted to the last severe stage of AD [7].

Magnetic Resonance Imaging (MRI) data has been popularly used in AD patient stratification due to MRI’s high-quality three-dimensional images of brain. Based on MRI data, regions of interest (ROI) which affect disease development could be revealed, contributing significantly to AD diagnosis and treatment. There are two main approaches based on ROIs, the single-ROI based approach [8] and the multiple-ROI based approach [9–11]. Chupin et al. [8] used probabilistic and anatomical priors for hippocampus segmentation to determine AD, NC, MCI. Ahmed et al. [11] proposed an automatic classification framework for AD, normal controls (NC), MCI, considering visual features from the most involved regions in AD. Several multivariate approaches, such as partial least squares and principal component analysis, were developed to build a discrimination model [12]. Liu et al. [13] constructed an individual network based on ROIs and used it as input of a classification model. Other previous work on multiple ROIs showed that they increased the performance of AD diagnosis [14]. In [15, 16], Liu et al. demonstrated not only ROIs, but also the correlations between ROIs were closely related to AD diagnosis results. Even though the previous methods have achieved remarkable results, they have not completely solved the problem of high dimensional data, in terms of accuracy and computational cost.

Cancer is not only threatening but also very diverse. Cancer patient stratification has been one of most challenging topics in biomedical informatics. Previous work have either focused on a specific data type of interest, such as gene expression, DNA methylation [17–20] or combined multiple data [21, 22]. Nowadays patient data has immensely been available with diverse information, such as gene expression, DNA methylation, miRNA expression, protein expression, exon expression, etc. [23]. It has been shown that there are multiple factors contributing to cancer pathology. Developing novel methods to combine a wide range of data is emerging and challenging [24–28].

The computation for learning high dimensional space is extremely complex and time-consuming. Since data types are of great difference (for example, categorial data, numerical data, imaging data), it is therefore essential to unify data measurement before integrating them. Lin et al. in [28] have proposed an effective method to combine data from different sources. They used multiple kernel learning to solve the second challenge and reduced data dimension basing on graph embedding. The method was applied to the image processing problem for ten data types in the Caltech-101 dataset [29]. In a recent study, Spacher et al. employed this method to cluster cancer patients and attained good results [30]. However, their work limited on the distribution of patients into different clusters and subtypes of cancers.

In this paper, we have proposed a novel computational framework based on fast multiple kernel learning and dimension reduction (fMKL-DR in short), addressing challenges in AD and cancer patient stratification. In the case study of AD patients, we enhanced significantly the performance of the multiple-ROIs approach when comparing with previous methods. Accuracy and Area Under a Curve (AUC) demonstrated that our proposed method was more accurate and robust than previous work. In the case study of cancer patients, the framework was applied to six types of cancers, i.e., glioblastoma multiforme cancer, ovarian cancer, lung cancer, breast cancer, kidney cancer, and liver cancer. We efficiently integrated gene expression, miRNA expression, and DNA methylation. The results showed that the classification model basing on integrated dataset was much more accurate than classification model basing on the single data type. The results obtained by both AD and cancer applications manifest that our developed model potentially stratifies patients and later aid disease prevention and prognosis.

## Methods

**Fig. 1:**
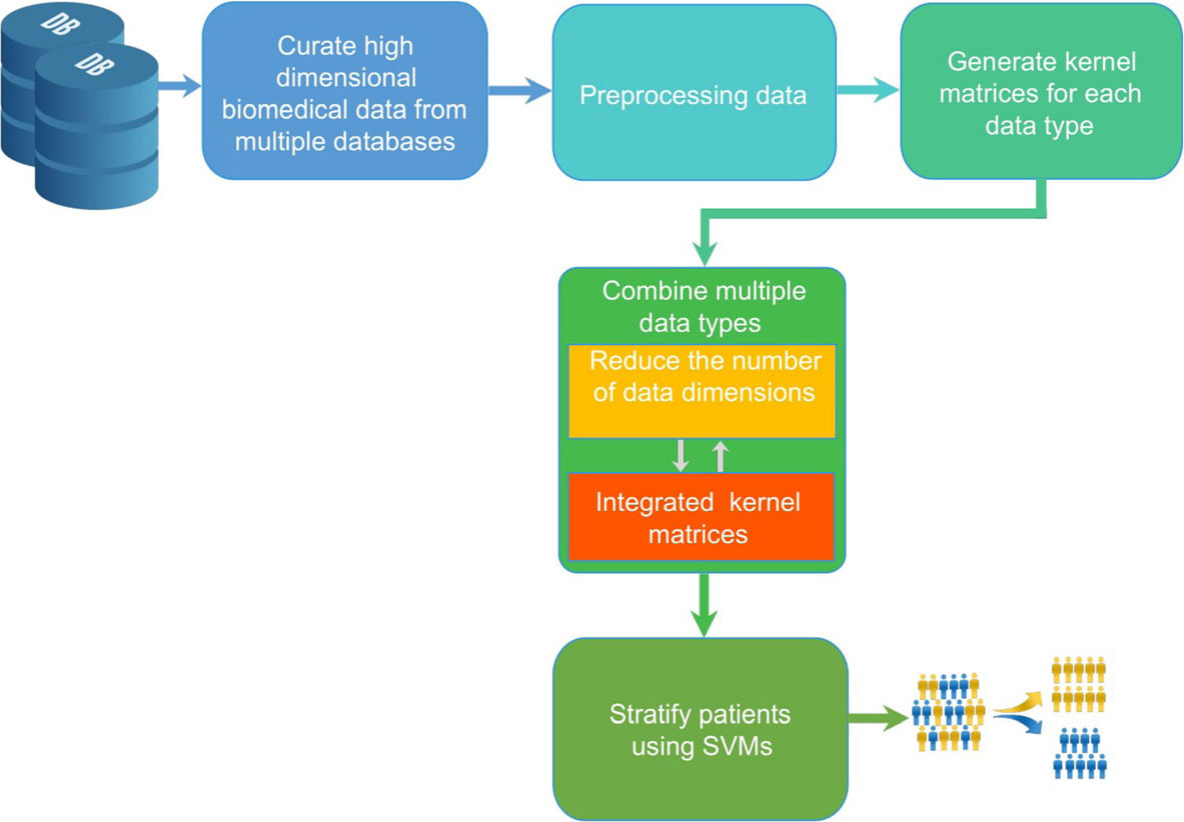
The systematic pipeline of the proposed method. Step 1 is to collect data from different data sources. Step 2 is to pre-processing data, removing noisy data, rescaling data, normalizing data. Step 3 is to generate kernel matrices and then the kernel matrices are then combined in the Step 4. The number of dimensions is also optimised to obtain the most suitable numbers of dimensions and reduce the computation cost. The last step is to stratify patients using SVMs

The proposed framework is demonstrated in Fig. 1. There are five main steps. In the first step, we obtained biological data related to cancer and AD from various information sources. These data in different types (e.g. imaging data, numerical data, textual data) were pre-processed in the second step. Data transformation is required to obtain a complete matrix, in which each row is a sample data and each column represents a feature. We employed a number of data pre-processing methods to eliminate redundant and noisy data. Furthermore, machine learning algorithms expect the scale of the training data to be equivalent, so we also used normalization to scale feature values to the range between − 1 and 1. We further employed a number of data pre-processing methods to eliminate redundant and noisy data. Specifically, we removed a feature if its data were missed in any subject (about 3% of the total number of features). Secondly, we generated kernel matrices from the above data matrix using different kernel functions. Each kernel matrix is a square symmetric and positive definite matrix, that represents the similarity of data samples based on a specific kernel function. In the next step, the kernel matrices are integrated into a final matrix using a multi-kernel learning framework. Since the kernel matrices have a large number of dimensions, we proposed the fast multi kernel learning framework combined with dimensional reduction algorithm, so-called fMKL-DR. Finally, we modelled predictive binary classifications with SVMs to stratify cancer and AD patients. Details of each step are described in the following sections.

### Data curation and pre-processing

In this section, we present the methods for collecting and pre-processing MRI images and genomics data for Alzheimer’s disease; proteomics and genomics data for cancer diseases.

### Image data from AD patients

MRI images of Alzheimer’s disease patients were extracted from Alzheimer’s Disease Neuroimaging Initiative (ADNI).^1^ The dataset consists of 710 T1-weighted subjects (data samples), including 200 subjects diagnosed with AD, 280 subjects with MCI, and 230 normal control (NC) subjects. Among 280 subjects with MCI, there are 120 subjects, that have MCI and convert to AD within 18 months (MCIc) and 160 subjects who have MCI and do not convert to AD (MCInc). To analyse the MRI images, we applied the six measures for cortical and sub-cortical regions proposed by Liu et al. [13]. More specifically, those six measures are Cortical Gray Matter Volume (CGMV), Cortical Thickness (CT), Cortical Surface Area (CSA), Cortical Curvature (CC), Cortical Folding Index (CFI) and Sub-cortical Volume (SV). We performed a complete procedure of image pre-processing, including spatial normalization, intensity normalization, skull stripping, segmentation and fill for obtaining the higher quality images. As the result, all images were registered with AAL atlas [31] before anatomic re-construction by FreeSurfer software.^2^

**Fig. 2:**
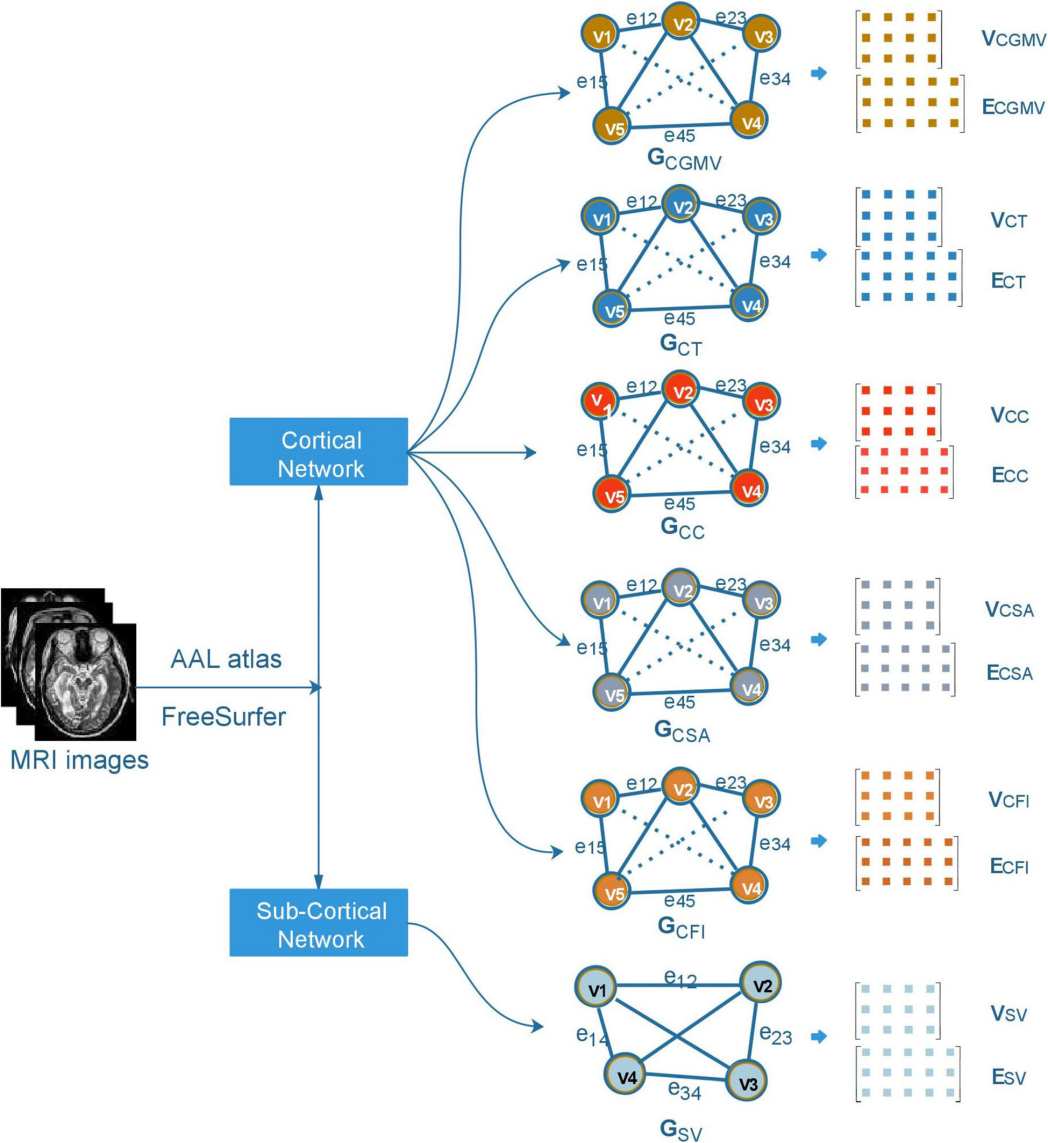
The illustration of the AD MRI images pre-processing. Firstly, we pre-processing MRI images by FreeSurfer software based on AAL atlas. Then, we construct six individual networks on cortical and subcortical areas from MRI images based on six anatomical measures, which included GCGMV, GCT, GCSA, GCC, GCFI, GSV. Each network includes two kinds of dataset: *V* and *E*

After calculating the six measures for cortical and sub-cortical regions, we represented the obtained data in terms of graphs. We generated six graphs *G*_*k*_ corresponding to six measures, namely as *G*_*CGMV*_, *G*_*CT*_, *G*_*CSA*_, *G*_*CC*_, *G*_*CFI*_, *G*_*SV*_. In a graph *G*_*k*_, the set *V*_*k*_ denotes set of vertices *(v*_*i*_*)*, representing the regions. We denote *E*_*k*_ as the set of edges *(e*_*ii*_*)*, consisting the weighted connections between two regions. The weight *w*_*ij*_ of the edge *e*_*ij*_ between two regions *i* and *j* is calculated as following:

$$ {w}_{ij}=\frac{1}{d_{ij}+1} $$

where *d*_*ij*_ is the distance between two regions and *d*_*ij*_ =  ∣ *m*_*i*_ − *m*_*j*_∣ (given *m*_*i*_ and *m*_*j*_ are measure values of region *i* and region *j,* respectively). For example, *G*_*CGMV*_ consists of 78 vertices and 3003 edges. In case of the sub-cortical region, G_SV_ was constructed by the set *V*_*i*_ of 12 vertices and the set *E*_*i*_ of 66 edges. Both *V*_*i*_ and set *E*_*i*_ are matrices *R[m,n]*, where *m* is the number of samples/subjects in our model, and *n* is the number of vertices or the number of edges, respectively. The illustration of the AD’s MRI images pre-processing is shown in Fig. 2.

### Genomic data from AD patients

In addition to MRI images, gene expression data of AD patients is very interesting for stratifying AD patients. From the ADNI database,^3^ we downloaded a raw gene expression dataset of 442 subjects, which includes 43 AD subjects, 139 MCI subjects, and 260 NC subjects. We extracted 22,609 genes for each subject and represented the gene expression dataset as a matrix, in which columns are subjects and rows are genes.

### Proteomic and genomic data from cancer patients

We extracted six cancer patient datasets from the TCGA database (The Cancer Genome Atlas, 2019)^4^ including Glioblastoma Multiforme (GBM), Ovarian Serous Cystadenocarcinoma (OV), Squamous Cell Lung Carcinoma (LUNG), Breast Invasive Carcinoma (BREAST), Kidney Renal Clear Cell Carcinoma (KIDNEY), and Liver Cancer (LIVER). In order to acquire multiple biomedical aspects related cancer, three data features were investigated in our model of cancer patient stratification, specifically gene expression, miRNA expression, DNA methylation. The statistics of cancer datasets are presented in Table 1. The raw data were pre-processed by removing the missing data and represented each data type as a matrix *R[m,n]*, in which columns are subjects, and rows are genes.

**Table 1:** Statistics of datasets in the cancer case study

Cancer	Number of Samples	Number of features (dimensions) per data type		
		Gene expression	DNA methylation	miRNA expression
LUNG	106	12,042	23,074	352
GBM	275	12,042	22,896	534
BREAST	435	12,042	24,978	354
OV	541	12,042	21,825	799
KIDNEY	122	17,899	24,960	329
LIVER	451	13,426	25,168	216

### Data rescaling

The values in each dataset have different magnitudes, units, and ranges. This variety causes an issue that the higher magnitude dataset will have greater weight than the lower ones. Therefore, we rescaled all of data values into the same range, enabling the equity between datasets. We used min-max scaling to scale the data into the range [− 1, 1].

### Multiple kernel learning combined with dimensionality reduction (MKL-DR)

**Fig. 3:**
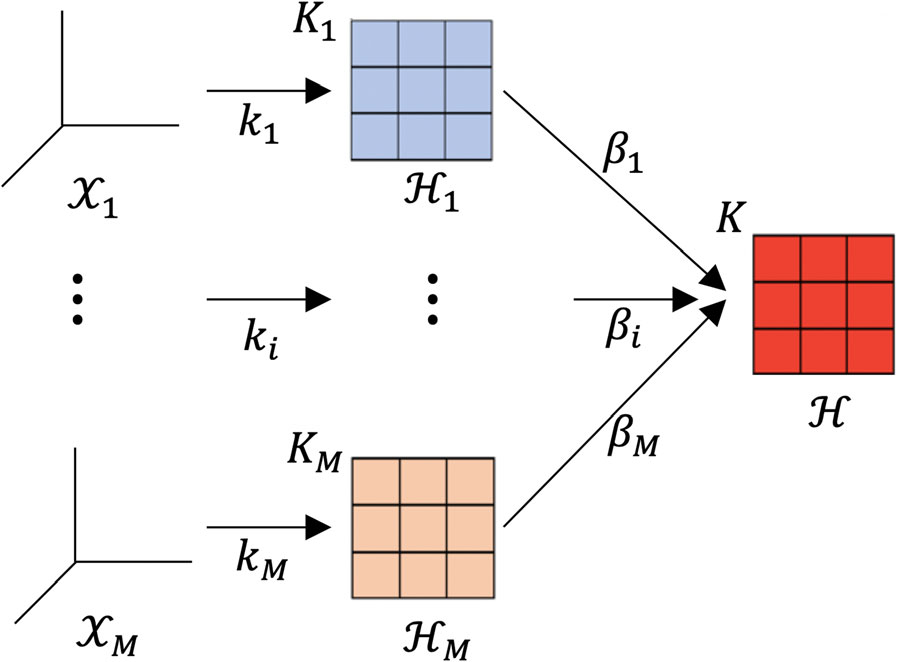
The illustration of Multiple Kernel Learning given that ${\mathcal{X}}_i$ is original dataset, *K*_*i*_ is kernel matrix that is constructed by kernel function *k*_*i*_, *β*_*i*_ is weighted coefficient combining *K*_1_ to *K*_*M*_ to unify a final kernel matrix *K*, ${\mathcal{H}}_i$ is the Hilbert space of *i*th dataset in the kernel method

Multiple Kernel Learning (MKL) is a machine learning method, modelling a kernel ensembled from many kernel functions or kernel matrices. Recent research on MKL have shown that learning SVMs with multiple kernels not only increases the accuracy but also enhances the expandability of the classification [25]. The MKL framework aims to the optimal for linear combination from input kernels. MKL’s illustration is shown in Fig. 3.

In Lin et al. [28], multiple kernel learning was improved by combining it with dimensionality reduction algorithm, so-called the MKL-DR. This framework was developed basing on graph embedding [32]. Yan et al. constructed an ensemble model, which enabled the incorporation of several dimensional reduction methods. The method presented data in the form of graph and provided a unified framework for a broad set of DR algorithms. Moreover, the paper developed a new dimensional reduction method. Based on the input graph, the rejection vector was found to project the vertices of graph in new low-dimensional space so that it best characterised the similar relationship between pairs of training samples basing on the graph preserving criterion. MKL-DR integrated better data from different sources and reduced the data dimensions, enhancing accuracy and computational cost. In this paper, we embedded Linear Discriminant Analysis [33] into the MKL-DR framework.

### Fast MKL-DR using dynamic programming

There are three parameters that affect the performance of the MKL-DR, i.e., the number of samples (*N*), the number of data types (*M*), and the dimensions after being reduced (*P*). In case that the value of *M* is small, often between 3~10, the number of dimensions after being reduced is small (in our experiment, we chose *P* = 5). Therefore, the computation complexity is *O*(*N*^3^), which is polynomial time. The MKL-DR training algorithm calculates iterative equations:

1 $$ {S}_W^A=\sum \limits_{i,j=1}^N{w}_{ij}{\left({\mathbbm{K}}^{(i)}-{\mathbbm{K}}^{(j)}\right)}^{\mathrm{T}}{AA}^{\mathrm{T}}\left({\mathbbm{K}}^{(i)}-{\mathbbm{K}}^{(j)}\right) $$

2 $$ {S}_{W\hbox{'}}^A=\sum \limits_{i,j=1}^Nw{\hbox{'}}_{ij}{\left({\mathbbm{K}}^{(i)}-{\mathbbm{K}}^{(j)}\right)}^{\mathrm{T}}{AA}^{\mathrm{T}}\left({\mathbbm{K}}^{(i)}-{\mathbbm{K}}^{(j)}\right) $$

3 $$ {S}_W^{\beta }=\sum \limits_{i,j=1}^N{w}_{ij}\left({\mathbbm{K}}^{(i)}-{\mathbbm{K}}^{(j)}\right){\beta \beta}^{\mathrm{T}}{\left({\mathbbm{K}}^{(i)}-{\mathbbm{K}}^{(j)}\right)}^{\mathrm{T}} $$

4 $$ {S}_{W\hbox{'}}^{\beta }=\sum \limits_{i,j=1}^Nw{\hbox{'}}_{ij}\left({\mathbbm{K}}^{(i)}-{\mathbbm{K}}^{(j)}\right){\beta \beta}^{\mathrm{T}}{\left({\mathbbm{K}}^{(i)}-{\mathbbm{K}}^{(j)}\right)}^{\mathrm{T}} $$

Given ${S}_W^A,{S}_{W\prime}^A,{S}_W^{\beta },{S}_{W\prime}^{\beta }$ are the matrices, which is used in the optimization problem [28]; *w*_*ij*_*, w’*_*ij*_ are elements of the similarity matrices aggregated from the kernel matrices; A is the sample coefficient matrix, and *β* is the kernel weight vector.

Each of four above Eqs. (1), (2), (3), (4) calculates the sum of the matrix chain multiplication (see more details in [28]). As the result, the MKL-DR will become exhaustively time-consuming when increasing the number of samples.

Matrix chain multiplication has a combinatory property, meaning that changing calculation order between a pair of the matrices will affect the number of product operations without modifying the multiplication results. Therefore, we propose to use a dynamic-programming-based procedure to find the calculation sequence that gives minimum product operations. If the number of product operations is minimum, the computation time of the equations will be reduced.

The minimum product operation order problem based on dynamic programming:

Given N matrices *A*_1_, *A*_2_, …, *A*_*N*_ with size of *A*_*i*_ is *d*_*i* − 1_ × *d*_*i*_.

*Problem*: Find the order of product matrix chain *A*_1_ × *A*_2_ × … × *A*_*N*_ to minimize the number of product operations.

*Solution*: Construct matrix *F* is a matrix *N* × *N*, with element *F*(*i*, *j*) is the total product operations to calculate matrix chain multiplication from *A*_*i*_ to *A*_*j*_ (*A*_*i*_ × *A*_*i* + 1_ × … × *A*_*j*_). The formula to calculate *F*(*i*, *j*) defined by:

$$ F\left(i,i\right)=0, $$

$$ F\left(i,i+1\right)={d}_{i-1}\times {d}_i\times {d}_{i+1}, $$

$$ F\left(i,j\right)=\mathit{\min}\ \left(F\left(i,t\right)+F\left(t+ 1,j\right)+{d}_{i\hbox{-} 1}\times {d}_t\times {d}_j\right) $$

with *t* = *i* + 1, *i* + 2, …, *j* − 1. In other words, *t* is a midpoint to insert the parentheses to change the calculation order so that the number of product operations is minimum:

$$ {A}_i\times {A}_{i+1}\times \dots \times {A}_j=\left({A}_i\times {A}_{i+1}\times \dots \times {A}_t\right)\left({A}_{t+1}\times {A}_{t+2}\times \dots \times {A}_j\right) $$

The dimension of the matrix of (*A*_*i*_ × *A*_*i* + 1_ × … × *A*_*t*_) is *d*_*i*–1_ × *d*_*t*_, and the dimension of the matrix of (*A*_*t* + 1_ × *A*_*t* + 2_ × … × *A*_*j*_) is *d*_*t*_ × *d*_*j*_.

Based on the above assumption, we developed an improved algorithm to matrix chain multiplication to minimize product operations shown in Algorithm 1.

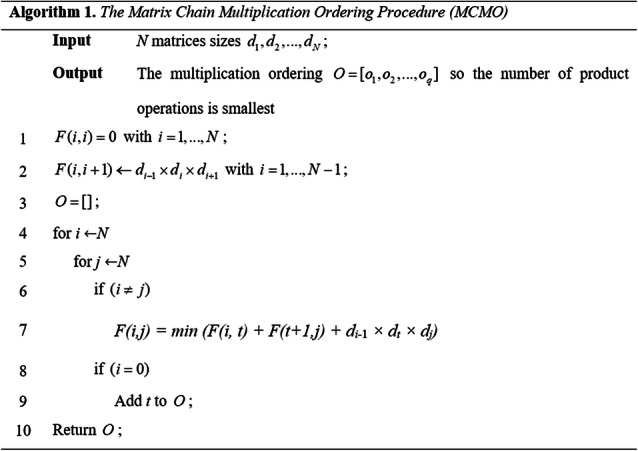



The MCMO complexity is *O*(*N*^3^). However, in the above equations, *N* (the number of matrices in chain) is small and equals to 4. Consequently, the time consumption of MCMO is trivial. Moreover, MCMO only calls 2 times in fMKL-DR, which is built as demonstrated in Algorithm 2.

The fast MKL-DR algorithm is described below.

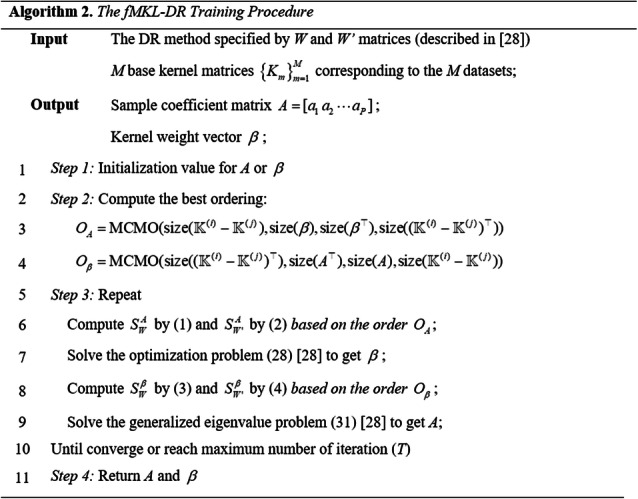



The matrices ${S}_W^A,{S}_{W\hbox{'}}^A,{S}_W^{\beta },{S}_{W\hbox{'}}^{\beta }$ are calculated in the lines 6th and 8th of Algorithm 2 based on the ordering of *O*_*A*_ and *O*_*β*_. These matrices have the same values as MKL-DR ones.

### Experimental design

We carried out three main experiments to investigate the performance of our method. The first experiment was done on the AD patients’ the MRI image dataset, comparing our proposed method to previous work. In the second experiment, we tested our method on the AD patients’ gene expressions. The last experiment was designed based on the six cancer patient datasets. In all of the experiments, we evaluated three measurements including accuracy, AUC, and computational time.

#### E1: Experiment based on MRI images dataset of Alzheimer’s disease patients

After pre-processing the Alzheimer’s disease patients MRI images datasets, we generated 12 individual networks. We designed the experiment as follows:

- Step 1. We built multiple kernel matrices by using different parameters from 12 original datasets. Specifically, from each dataset, we used the Gaussian kernel function *k*(*x*, *x*') = exp(−‖*x* − *x*'‖^2^/2*σ*^2^). We run the experiments with 5 different parameters *σ*∈ {10^− 6^, 10^− 3^, 1, 10^3^, 10^6^} to generate 5 kernel matrices. We constructed 60 kernel matrices from 12 original datasets, that were used as input data for the next step.
- Step 2. Develop four models for the four binary classification problems, AD and NC, AD and MCI, NC and MCI, and MCIc and MCInc, denoted by C_AD/NC_, C_AD/MCI_, C_NC/MCI_, and C_MCIc/MCInc_ correspondingly. We employed libSVM library^5^ with 60 kernel matrices generated in Step 1. The set of parameters is the default one in libSVM with -s (svm_type) = 0 (C-SVC), −c (cost) = 1, −wi (weight) = 1.
- Step 3. For each classification problems (C_AD/NC_, C_AD/MCI_, C_NC/MCI_, and C_MCIc/MCInc_), 20 experiments were performed. In each experiments, 2/3 dataset were randomly selected from the original dataset for training, and the rest was used for testing. The best result among the 20 experiments is reported and statistically tested.
- Step 4. Compare the results between our proposed method and the recent other methods.

#### E2: Experiment based on Alzheimer’s disease patient gene expression dataset

We designed the experiment based on AD patient gene expression dataset as follows:

- Step 1. Generate four different kernel matrices by four different kernel functions including Gaussians, Polynomial, Linear, Sigmoid kernel function, which were developed in the dimensionality reduction library.^6^ Default parameters were set as below.
  - Gaussian Kernel: t is number of samples.
  - Polynomial: d = 2
  - Linear: c = 0
  - Sigmoid: α = 1/D, D is the number of dimensions of dataset, and c = 0.
- Step 2. Integrate and reduce dimensions from the four kernel matrices from Step 1 by fMKL-DR into a unified kernel.
- Step 3. Develop five classification models based on the libSVM library, i.e., C_Gaussian_ with Gaussian kernel matrix), C_Polinomial_ with Polinomial kernel matrix), C_Linear_ with Linear kernel matrix), C_Sigmoid_ with Sigmoid kernel matrix), and C_fMKL-DR_ with unified kernel matrix that is got in Step 2). The libSVM parameters were set as default values (showed in Step 1 of E1).
- Step 4. Carry out the same procedure as Step 3 in E1.
- Step 5. Compare the results between the classification model using single gene expression dataset with different kernel functions (Step 1) and the unified kernel (Step 2).

#### E3: Experiment based on cancer patient datasets

We carried out the following four steps on each dataset to evaluate the proposed method.

- Step 1. Run the fMKL-DR algorithms by initializing A or *β*. Both of initializations produce similar results, even though the first initializing A obtains faster convergence. Integrate multiple data sources and reduce the number of data dimensions by fMKL-DR.
- Step 2. Develop four classification models based on three single datasets and one combined dataset and SVMs, i.e., C_GE_ for the gene expression dataset, C_DNA_ for the DNA methylation dataset, C_miRNA_ for the mirRNA expression dataset), and C_fMKL-DR_ for the unified kernel matrix obtained in Step 1. For the three models (C_GE_, C_DNA_, and C_miRNA_), we used the default values in libSVM with Gaussian kernel function (gamma = 1/num_features, num_features is the number of features of data). In the case of the model C_fMKL-DR_, the kernel is the one obtained unified kernel matrix and the other parameters are set as the default values in libSVMs.
- Step 3. Perform the same procedure as Step 3 in E1.
- Step 4. Compare the results between the classification model using a single data type and the one with data integration.

These abovementioned experimental steps were run on the six patient datasets of cancer patients to evaluate the efficiency of the methods.

### Statistical test

We performed statistical tests to assess the robustness of the obtained results, specifically one sample *t-test* with *n* = 20. At 95% confidence level, the hypothesis tests (on mean of accuracy and AUC) were done to evaluate whether they are statistically significant.

## Results

### Application of stratifying Alzheimer’s disease patients

To investigate performance of our classification model, we carried out four experiments for four classification subgroups including AD/NC, AD/MCI, NC/MCI, MCIc/MCInc (MCI and converted to AD/ MCI and not converted to AD). We evaluated our method by comparing accuracy and area under curve (AUC) of our classification model to previous works, which used the same dataset of the MRI images from ADNI.

Tables 2 and 3 showed accuracy and AUC of the previous work and our method applied to the four patient subgroups, respectively. In term of both accuracy and AUC, the whole brain-based method achieved significantly better results than single ROI-based method [8] or multiple ROIs-based method proposed by [11]. The results showed that the multiple ROIs-based approach was more appropriate than the other. It is well aligned with clinical practice that AD related to all of brain regions, rather than a specific one or some regions. In comparison with the whole brain-based methods proposed by Khedher et al. [12], and Suk et al. [10], and Dai et al. [9], or Liu et al. [13], we obtained better accuracy and AUC than they did. AUC of our proposed method is close to 1, demonstrating the accurateness and robustness of the method.

**Table 2:** Accuracy of the previous methods and the proposed method for different AD patient groups

Method	Classification Model Accuracy (%)			
	AD/NC	AD/MCI	NC/MCI	MCIc/MCInc
Chupin et al., 2009 [8]	80.51	73.48	71.94	64.21
Ahmed et al., 2015 [11]	86.40	74.51	76.29	68.72
Khedher et al., 2015 [12]	88.96	84.59	82.41	70.11
Dai et al., 2013 [9]	90.81	85.92	81.92	71.04
Suk et al., 2014 [10]	93.05	88.98	83.67	72.86
Liu et al., 2018 [13]	95.24	90.85	86.35	74.28
Proposed method (the best result among 20 runs)	96.50	91.25	87.65	78.49
Proposed method (at 90% confidence level of *t-test*)	95.80	90.63	86.47	77.42

**Table 3:** Comparative AUC results of the previous methods and the proposed method for different AD patient groups

Method	AUC			
	AD/NC	AD/MCI	NC/MCI	MCIc/MCInc
Chupin et al., 2009 [8]	0.7851	0.7328	0.7155	0.6638
Ahmed et al., 2015 [11]	0.8487	0.7562	0.7677	0.6814
Khedher et al., 2015 [12]	0.9256	0.8859	0.8134	0.7076
Dai et al., 2013 [9]	0.9429	0.8743	0.8118	0.7086
Suk et al., 2014 [10]	0.9475	0.9007	0.8203	0.7123
Liu et al., 2017 [13]	0.9754	0.9355	0.9107	0.7885
Proposed method (the best result among 20 runs)	0.9786	0.9412	0.9151	0.8024
Proposed method (at 90% confidence level of *t-test*)	0.9705	0.936	0.911	0.7945

**Fig. 4:**
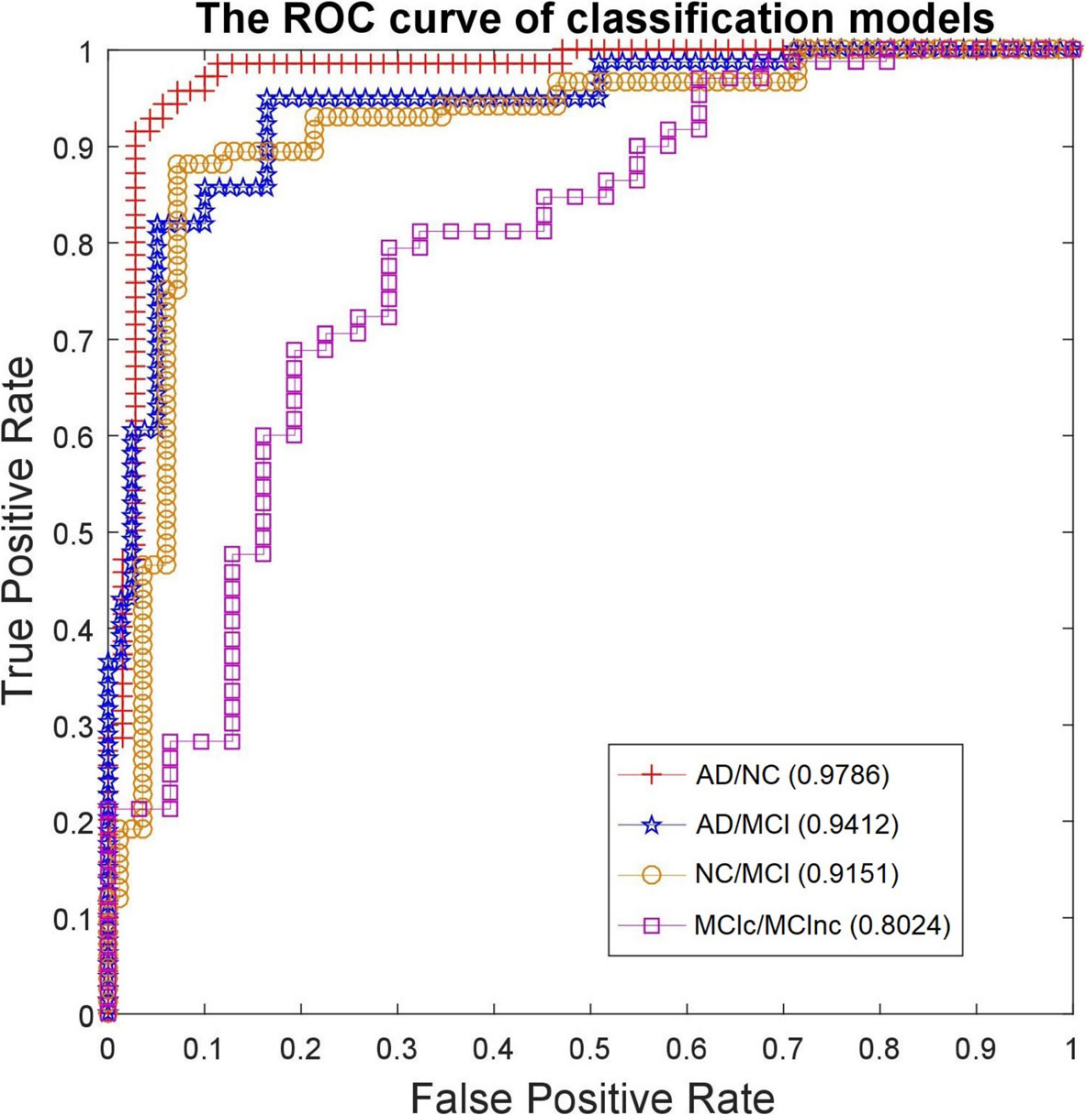
The four ROC curves of the classification models on the MRI data of AD patients

Figure 4 shows the ROC curves of four analysis groups. We achieved high AUCs for all of the groups. The AD/NC model had the highest value equal to 0.978, followed by AD/MCI equal to 0.941, NC/MCI equal to 0.915, and MCIc/MCInc equal to 0.802. The ROC confirmed that our method was highly efficient in stratifying AD patients for all phases.

Gene expression data of the AD patients was processed and run with different kernels functions in our framework, specifically *k1* = Gaussians, *k2* = Polynomial, *k3* = Linear, *k4* = Sigmoid, and our integrated kernel function (see more in Table 4). Accuracy of the integrated kernel function method was higher than all of single-kernel function methods on the same dataset.

**Table 4:** Case study of the AD patient stratification, accuracy between gene expression and proposed method integrated four kernels (different kernel functions: k1 = Gaussians, k2 = Polynomial, k3 = Linear, k4 = Sigmoid). fMKL-DR is the best accuracy among the 20 runs, and fMKL-DR^a^ is accuracy tested at 95% confidence level

Tasks	# Subjects	Accuracy (%)					
		Gaussian	Polynomial	Linear	Sigmoid	fMKL-DR	fMKL-DR^a^
AD/NC	303	88.12	87.13	88.19	87.13	91.09	90.1
AD/MCI	182	83.33	81.67	83.33	80.00	85.00	83.33
NC/MCI	399	70.68	69.92	69.92	69.17	75.94	75.19

### Application of stratifying cancer patients

**Fig. 5:**
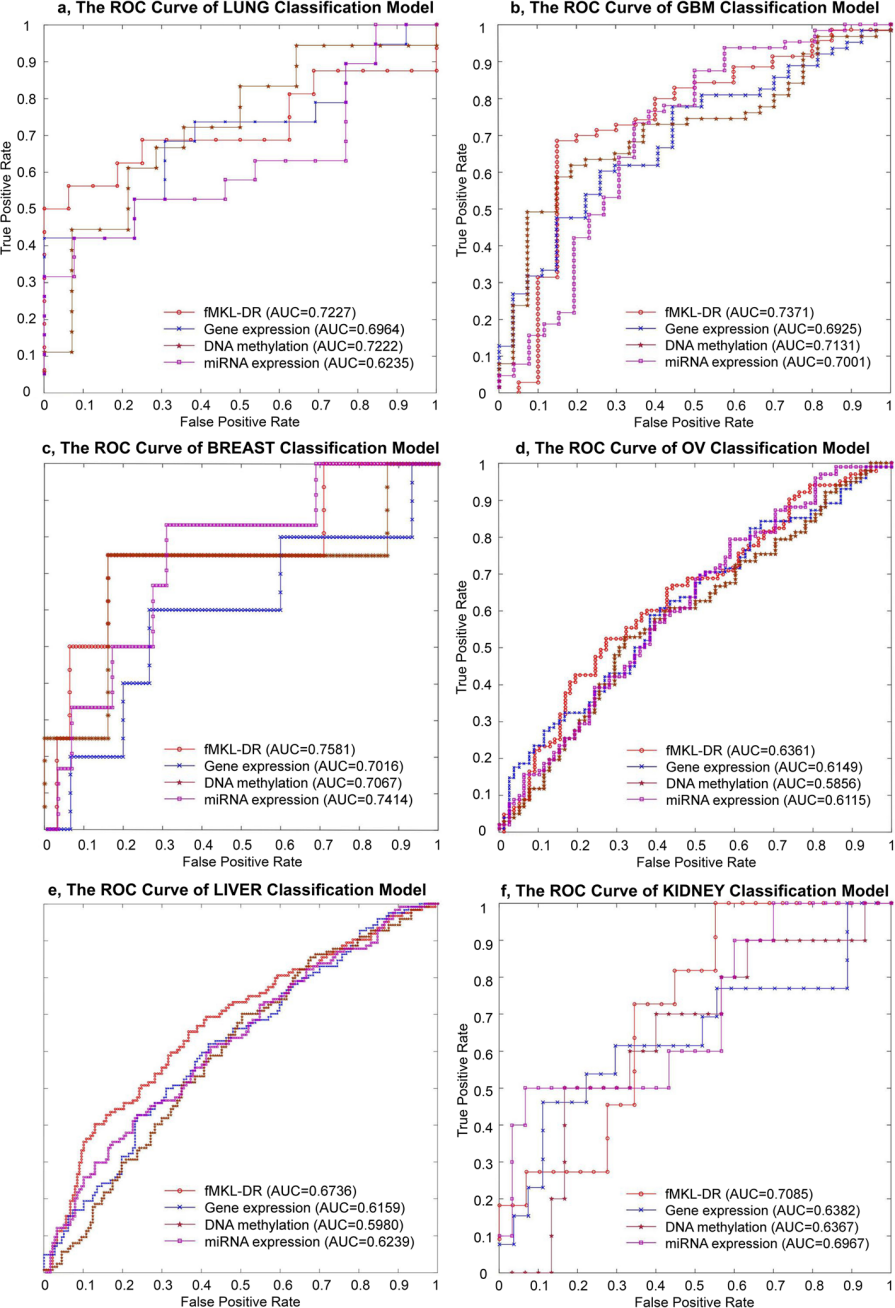
The ROC curves of the classifiers for the six cancer datasets

The accuracy and reliability of classification models is shown in Fig. 5 and Table 5. The results demonstrated that classification models based on a single data type were less accurate than the one basing on integrated data. Specifically, testing the method on all cancer datasets, the fMKL-DR model produced the best accuracy.

**Table 5:** Case study of the cancer patient stratification: accuracy obtained for each dataset and data integration. fMKL-DR is the best accuracy among the 20 runs, and fMKL-DR^a^ is accuracy tested at 95% confidence level

Cancer	Number of Samples	Alive/Dead	Gene expression	DNA methylation	miRNA expression	fMKL-DR	fMKL-DR^a^
LUNG	106	42/64	62.50	65.63	71.88	78.13	75.00
GBM	275	202/73	77.44	75.56	76.67	81.11	80.00
BREAST	435	360/75	88.57	88.57	91.43	94.29	93.57
OV	541	258/283	59.44	58.33	55.00	62.22	61.67
KIDNEY	122	90/32	81.25	81.25	78.13	87.50	85.00
LIVER	451	277/174	66.00	69.00	65.00	72.00	71.33

Table 5 shows accuracy of the classifiers on the different datasets. In this table, the fourth, fifth, and sixth columns represent accuracy of the classifiers based on single datasets, such as Gene expression, DNA methylation, and miRNA expression. The last two columns show accuracy of the classifier based on the integrated dataset with fMKL-DR. The classifier on the integrated dataset has a high accuracy ranging from ~ 72% to ~ 94%. Especially, accuracy on BREAST and KIDNEY datasets were very high, values of 94.29 and 87.50%, respectively. Thus, in all cancer patient datasets, the classifiers based on fMKL-DR have demonstrated to be effective for patient stratification since it obtained better results than the other classifiers based on each single dataset.

Figure 5 shows the ROC curve of the classifiers on the different cancer datasets, including lung cancer, GBM, breast cancer, OV cancer, liver cancer, and kidney cancer. For each data type of cancers, we drew four ROC curves corresponding to the four classifiers training on Gene Expression, DNA Methylation, miRNA Expression and the integrated dataset. The classifier implemented by our method (fMKL-DR) obtained the best AUC value when compared to classifiers on each individual dataset. In addition, these AUC values are relatively high, ranging from 0.63 to 0.75, this implies that the predicted results of our models are reliable.

## Discussion

The fMKL-DR framework has been showed the robustness and accurateness in both applications of AD and cancer patient stratification. We have tested different datasets of heterogenous data types, from imaging data to numerical data. The numbers of dimensions are also varied. The classification models were built for various cancer types and all of them achieved significant results, showing the high generalization of the model. When stratifying AD patients, the method could classify even the different phases of AD patients, not only AD or non-AD patients. Therefore, it is very promising for early diagnosis and follow up of effective treatment for AD patients, especially since the late phase of AD is untreatable.

**Fig. 6:**
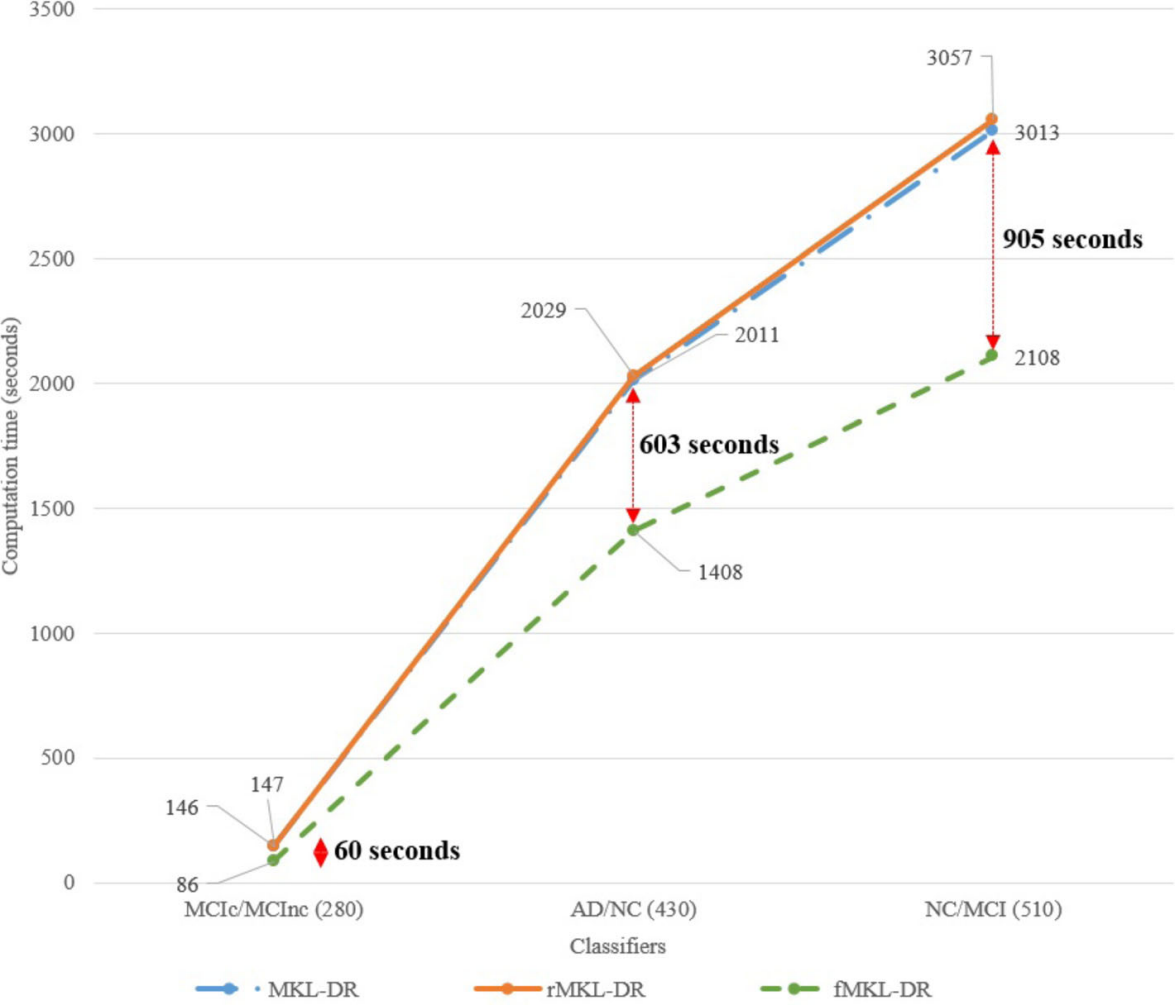
Computational time of the methods for the AD patient stratification with the different sizes of samples (iterations equals 10)

**Fig. 7:**
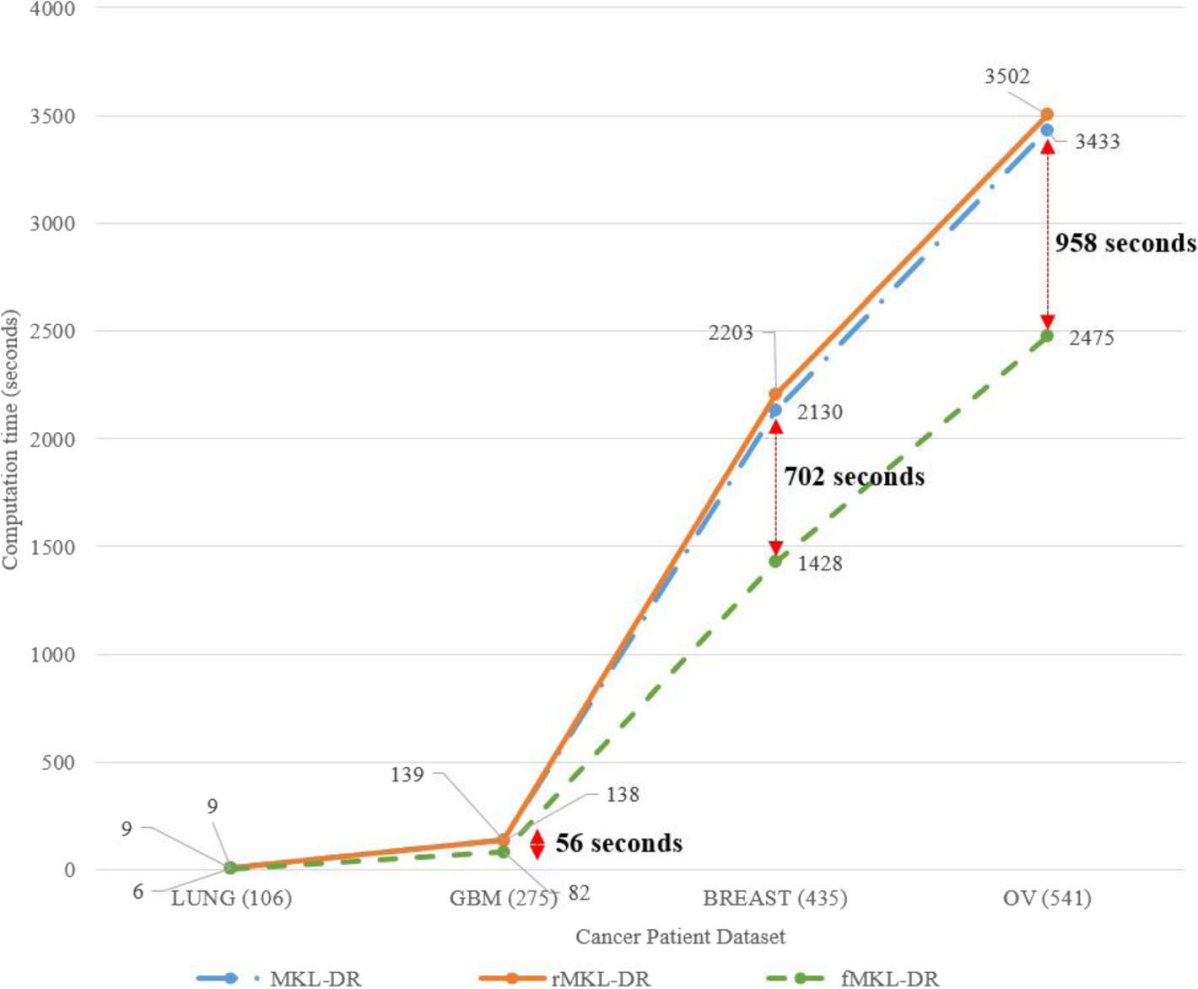
Computational time of the methods for the cancer patient stratification with the different sizes of samples (iterations equals 10)

In addition to high accuracy for both cancer and AD, our proposed method is much faster than the previous work. This advantage is of great significance because there are more and more data available, and data will be more and more complex. The problems of data sparsity, and heterogeneity requires cost-effective and accurate methods. Figure 6 and Table 6 show the computation time of two previous methods, MKL-DR, rMKL-DR and the proposed method fMKL-DR on Alzheimer’s Disease dataset. Figure 7 and Table 7 demonstrate the computational time when running the experiments on the cancer datasets. In all cases, our method reduced notably the computational cost. Especially, we saved more time than previous method did when the scales of datasets were increased. For example, we saved 702 s for the breast cancer dataset of 435 subjects, and 958 s for the ovarian cancer dataset of 541 subjects. Our proposed method is very beneficial when analysing huge data cohorts.

**Table 6:** The comparison of the computational time of MKL-DR, rMKL-DR and the proposed method (in bold) on Alzheimer’s Disease MRI dataset

Tasks	#Samples	Computation time (seconds)								
		5 iterations			10 iterations			20 iterations		
		MKL-DR	rMKL-DR	fMKL-DR	MKL-DR	rMKL-DR	fMKL-DR	MKL-DR	rMKL-DR	fMKL-DR
AD/NC	430	1005	1014	**702**	2011	2029	**1408**	4022	4060	**2817**
AD/MCI	480	1322	1337	**843**	1646	1675	**1687**	3292	3351	**3374**
NC/MCI	510	1506	1528	**996**	3013	3057	**2108**	6027	6115	**4217**
MCIc/MCInc	280	73	73	**43**	146	147	**86**	293	294	**173**

**Table 7:** Computation time (in second) in comparing to previous methods for the cancer datasets

Cancer	Num of Samples	Computation time (seconds)								
		5 iterations			10 iterations			20 iterations		
		MKL-DR	rMKL-DR	fMKL-DR	MKL-DR	rMKL-DR	fMKL-DR	MKL-DR	rMKL-DR	fMKL-DR
LUNG	106	4	4	**3**	9	9	**6**	18	19	**13**
KIDNEY	122	5	5	**4**	12	12	**8**	36	37	**27**
GBM	275	69	69	**41**	138	139	**82**	276	279	**165**
BREAST	435	1064	1074	**714**	2130	2149	**1428**	4262	4298	**2857**
LIVER	451	1183	1195	**752**	2367	2391	**1508**	4734	4782	**3017**
OV	541	1716	1750	**1237**	3433	3502	**2475**	6867	7005	**4952**

**Fig. 8:**
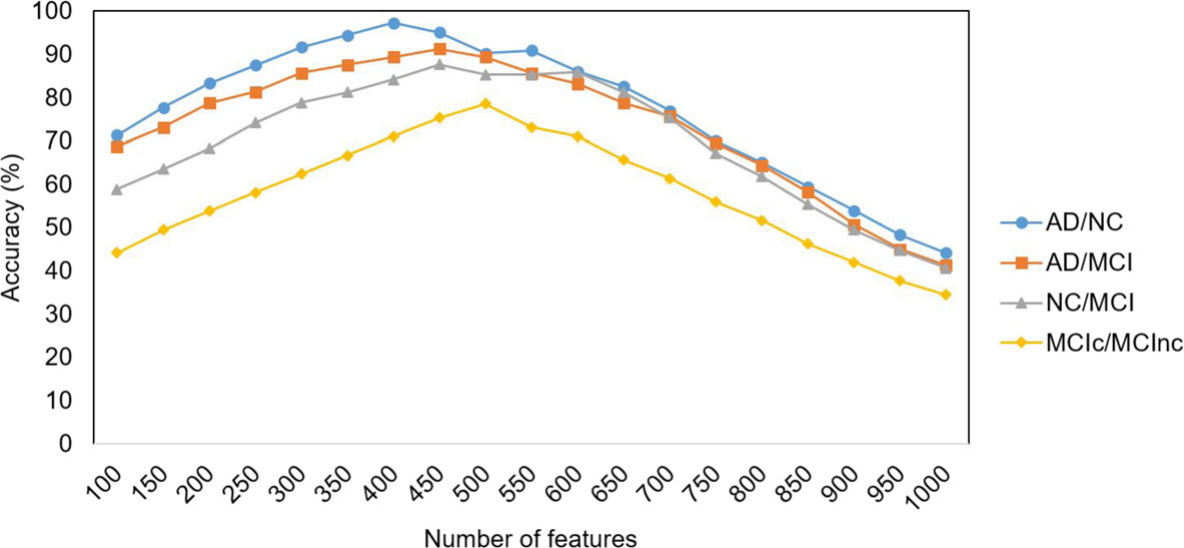
The accuracy of the models with different numbers of reduced dimensions for AD/NC, AD/MCI, NC/MCI, and MCIc/MCInc classification

The fMKL-DR method is advantageous in optimizing the best number of dimensions/features after reduction, denoted as *f*. Feature selection and feature engineering are crucial steps to acquire the best performance. We set *f* from 100 to 1000 by step of 50, and run all the tests with set values of *f*. The results of AD patient stratification with different numbers of features are showed in Fig. 8. The highest accuracy is obtained with about 400 features for AD/NC classification, about 450 AD/MCI and NC/MCI classifications, and about 500 features for MCIc/MCInc classification. It turns out two main points. Firstly, the optimal number of features is classifier-independent, meaning that there is no common set up of dimension reduction for all classifiers. Secondly, increasing number of dimensions does not always improve performance of the model, even decrease accuracy. As the result, our framework optimised the numbers of dimensions to ensure the best performance of the classifier.

There are some limitations that will be improved in the future work. Firstly, data preprocessing, including missing data handling, will be better performed. In this paper, we removed a feature if its data were missed in any subject. Therefore about 3% of the total number of features (e.g., 20,000 genes of the whole genome) were removed. The missing mechanism will be deeply analysed to define the type of missing data, missing completely at random or missing at random or missing not at random. Depending on the types of missing data, a appropriate technique, such as data imputation, will be applied. Secondly, parameter optimisation will be of interest. We set the default values in our experiments to ensure the comparative evaluation with the other related work, however the better performance can be obtained by testing several sets of parameters and parameter optimisation algorithms.

The proposed framework is very useful when analysing high dimensional genomics data. Dimension reduction can be applied in the significance analysis on gene expression data. In multi-assay data exploration, dimension reduction facilitates downstream gene set, pathway and network analysis of variables. Integrative data analysis is of great interest due to the complexity of biology and medicine. Because multi-omics data, such as DNA sequence, epigenome, transcriptome, protein, metabolites, is more and more available, there is an increasing need for developing methods with high performance. Single kernel methods are not suitable to encode heterogenous or multi-modal datasets. The proposed framework can be easily adjusted to ingrate new data types, making it flexible in other applications, such as biomarker identification. The framework is adaptable for other disease studies, in addition to cancer and AD. Precision medicine is required big data integration, especially electronic health records and whole genome sequencing data. Therefore, our method will be very beneficial to precision medicine.

## Conclusions

In this paper, we have proposed the accurate and fast kernel learning framework. We employed the framework to stratify AD patients and cancer patients. We handled a wide range of data, including the MRI image data for AD, the gene expression data for both AD and cancer, miRNA expression, DNA methylation for cancer. By carrying out a number of testing strategies, the results showed that our model performed better than previous work, in term of accuracy, AUC, and computational time. In fact, the electronic health records will be more and more available, offering better insights of patient studies. As there are more and more data available from diverse sources, it is emerging to develop computational method to combine and mine those heterogenous data. Our proposed framework is very promising to handle high dimensional data and to aid precision medicine.

## Data Availability

The datasets were curated from public databases, ADNI (https://adni.loni.usc.edu), and TCGA Research Network (https://www.cancer.gov/tcga). The processed data along with codes are available upon request.

## Abbreviations

fMKL-DR: Fast Multiple Kernel Learning Framework
AD: Alzheimer’s disease
MCI: Mild Cognitive Impairment
ROI: Regions of Interest
MRI: Magnetic Resonance Imaging
NC: Normal Control
AUC: Area Under a Curve
MCIc: MCI and converted to AD
MCInc: MCI and not converted to AD
GBM: Glioblastoma Multiforme
CGMV: Cortical Gray Matter Volume
CT: Cortical Thickness CT
CSA: Cortical Surface Area
CC: Cortical Curvature
CFI: Cortical Folding Index
SV: Sub-cortical Volume
OV: Ovarian Serous Cystadenocarcinoma
MKL: Multiple Kernel Learning
MKL-DR: Multiple Kernel Learning and Dimensionality Reduction
MCMO: Matrix Chain Multiplication Ordering Procedure

## Footnotes

1 http://adni.loni.usc.edu

2 https://surfer.nmr.mgh.harvard.edu

3 http://adni.loni.usc.edu

4 https://www.cancer.gov/tcga

5 https://www.csie.ntu.edu.tw/~cjlin/libsvm/

6 http://www.cad.zju.edu.cn/home/dengcai/Data/data.html
